# Effect of tofogliflozin on arterial stiffness in patients with type 2 diabetes: prespecified sub-analysis of the prospective, randomized, open-label, parallel-group comparative UTOPIA trial

**DOI:** 10.1186/s12933-020-01206-1

**Published:** 2021-01-04

**Authors:** Naoto Katakami, Tomoya Mita, Hidenori Yoshii, Toshihiko Shiraiwa, Tetsuyuki Yasuda, Yosuke Okada, Keiichi Torimoto, Yutaka Umayahara, Hideaki Kaneto, Takeshi Osonoi, Tsunehiko Yamamoto, Nobuichi Kuribayashi, Kazuhisa Maeda, Hiroki Yokoyama, Keisuke Kosugi, Kentaro Ohtoshi, Isao Hayashi, Satoru Sumitani, Mamiko Tsugawa, Kayoko Ryomoto, Hideki Taki, Tadashi Nakamura, Satoshi Kawashima, Yasunori Sato, Hirotaka Watada, Iichiro Shimomura, On behalf of the UTOPIA study investigators

**Affiliations:** 1 Department of Metabolic Medicine, Osaka University Graduate School of Medicine, 2-2, Yamadaoka, Suita, Osaka, 565-0871 Japan; 2 Department of Metabolism and Atherosclerosis, Osaka University Graduate School of Medicine, 2-2, Yamadaoka, Suita, Osaka, 565-0871 Japan; 3 Department of Metabolism & Endocrinology, Juntendo University Graduate School of Medicine, Hongo 2-1-1, Bunkyo-ku, Tokyo, 113-8421 Japan; 4 Department of Medicine, Diabetology & Endocrinology, Juntendo Tokyo Koto Geriatric Medical Center, Koto-ku, Tokyo, 136-0075 Japan; 5 Shiraiwa Medical Clinic, 4-10-24 Hozenji, Kashiwara, Osaka 582-0005 Japan; 6 Department of Diabetes and Endocrinology, Osaka Police Hospital, 10-31, Kitayama-cho, Tennoji-ku, Osaka, 543-0035 Japan; 7 First Department of Internal Medicine, School of Medicine, University of Occupational and Environmental Health, Japan, 1-1, Iseigaoka, Yahatanishi-ku, Kitakyushu, 807-8555 Japan; 8 Department of Diabetes and Endocrinology, Osaka General Medical Center, 3-1-56, Bandai-Higashi, Sumiyoshi-ku, Osaka, 558-8558 Japan; 9 Department of Diabetes, Endocrinology and Metabolism, Kawasaki Medical School, 577 Matsushima, Kurashiki, Okayama 701-0192 Japan; 10 Nakakinen Clinic, 745-5, Nakadai, Naka, Ibaraki 311-0113 Japan; 11 Diabetes and Endocrinology, Kansai Rosai Hospital, 3-1-69, Inabaso, Amagasaki, Hyogo Japan; 12 Misaki Naika Clinic, 6-44-9, Futawa-higashi, Funabashi, Chiba Japan; 13 Kitasenri Maeda Clinic, 4-119, Furuedai, Suita, Osaka, 565-0874 Japan; 14 Jiyugaoka Medical Clinic, West 6, South 6-4-3, Obihiro, Hokkaido 080-0016 Japan; 15 Kosugi Medical Clinic, 3-9, Tamatsukurimoto-cho, Tennoji-ku, Osaka, 543-0014 Japan; 16 Otoshi Medical Clinic, 8-47, Kakudacho, Osaka Kita-ku, Osaka, 530-0017 Japan; 17 Hayashi Clinic, 3-9-23, Koshienguchi, Nishinomiya, Hyogo 663-8113 Japan; 18 Center for Diabetes and Endocrinology, Nippon Life Hospital, 2-1-54 Enokojima, Nishi-ku, Osaka, 550-0006 Japan; 19 Department of Endocrinology and Metabolism, Ikeda Municipal Hospital, 3-1-18, Jonan, Ikeda, Osaka 563-8510 Japan; 20 Center for Diabetes Mellitus, Osaka Rosai Hospital, 1179-3 Nagasone-cho, Kita-ku, Sakai, Osaka 591-8025 Japan; 21 Diabetes Center, National Hospital Organization Osaka National Hospital, 2-1-14, Hoenzaka, Chuo-ku, Osaka, 540-0006 Japan; 22 Department of Internal Medicine, Kawasaki Hospital, 3-3-1, Higashiyamacho, Hyogo-ku, Kobe, Hyogo 652-0042 Japan; 23 Kanda Naika Clinic, 5-21-3, Hannancho, Osaka Abeno-ku, Osaka, 545-0021 Japan; 24 Department of Preventive Medicine and Public Health, Keio University School of Medicine, 45 Shinanomachi Shinjuku-ku, Tokyo, 160-8582 Japan

**Keywords:** Arterial stiffness, Pulse wave velocity, Arteriosclerosis, Diabetes, SGLT2 inhibitor, Tofogliflozin

## Abstract

**Background:**

Tofogliflozin, an SGLT2 inhibitor, is associated with favorable metabolic effects, including improved glycemic control and serum lipid profile and decreased body weight, visceral adipose tissue, and blood pressure (BP). This study evaluated the effects of tofogliflozin on the brachial-ankle pulse wave velocity (baPWV) in patients with type 2 diabetes (T2DM) without a history of apparent cardiovascular disease.

**Methods:**

The using tofogliflozin for possible better intervention against atherosclerosis for type 2 diabetes patients (UTOPIA) trial is a prospective, randomized, open-label, multicenter, parallel-group, comparative study. As one of the prespecified secondary outcomes, changes in baPWV over 104 weeks were evaluated in 154 individuals (80 in the tofogliflozin group and 74 in the conventional treatment group) who completed baPWV measurement at baseline.

**Results:**

In a mixed-effects model, the progression in the right, left, and mean baPWV over 104 weeks was significantly attenuated with tofogliflozin compared to that with conventional treatment (– 109.3 [– 184.3, – 34.3] (mean change [95% CI] cm/s, *p* = 0.005; – 98.3 [– 172.6, – 24.1] cm/s, *p* = 0.010; – 104.7 [– 177.0, – 32.4] cm/s, *p* = 0.005, respectively). Similar findings were obtained even after adjusting the mixed-effects models for traditional cardiovascular risk factors, including body mass index (BMI), glycated hemoglobin (HbA1c), total cholesterol, high-density lipoprotein (HDL)-cholesterol, triglyceride, systolic blood pressure (SBP), hypertension, smoking, and/or administration of drugs, including hypoglycemic agents, antihypertensive agents, statins, and anti-platelets, at baseline. The findings of the analysis of covariance (ANCOVA) models, which included the treatment group, baseline baPWV, and traditional cardiovascular risk factors, resembled those generated by the mixed-effects models.

**Conclusions:**

Tofogliflozin significantly inhibited the increased baPWV in patients with T2DM without a history of apparent cardiovascular disease, suggesting that tofogliflozin suppressed the progression of arterial stiffness.

*Trial Registration* UMIN000017607. Registered 18 May 2015. (https://www.umin.ac.jp/icdr/index.html)

## Background

Sodium-glucose cotransporter 2 (SGLT2) inhibitors, a class of antidiabetic agents, have a pleiotropic effect, and thus diminish several cardiovascular risk factors by reducing visceral adipose tissue, body weight, and blood pressure (BP); improving the blood lipid profile; and generating a renoprotective effect independent of glycemic effects [1, 2]. Previous clinical trials have shown that SGLT2 inhibitors significantly reduced major cardiovascular (CV) adverse events and/or hospitalization for heart failure in patients with type 2 diabetes mellitus (T2DM) at high risk of cardiovascular disease (CVD) [3–5]. In addition, a recent meta-analysis indicated that SGLT2 inhibitors have robust benefits in reducing hospitalization for heart failure and progression of renal disease, regardless of existing atherosclerotic CVD or a history of heart failure [6]. These results suggest that SGLT2 inhibitors exert a broad range of beneficial effects on the CV system through multiple mechanisms.

Reduction in the arterial wall elasticity increases the vascular wall stress, resulting in the progression of atherosclerosis, an increase in the left ventricular afterload, left ventricular diastolic dysfunction, and coronary perfusion disorder. Moreover, deterioration in arterial stiffness was associated with hospitalization for new-onset heart failure in asymptomatic patients with CV risk factors [7]. Thus, arterial stiffness may reflect not only atherosclerotic changes but also risks of an extensive range of CVDs including cardiac dysfunction.

Previous studies have evaluated the effect of SGLT2 inhibitors on arterial stiffness using several indices [8–19], some of which indicated the beneficial effect of SGLT inhibitors. Pulse wave velocity (PWV) is commonly used to measure the severity of arterial stiffness, showing the structural and functional properties of the arterial wall [20, 21]. However, evidence on the effect of SGLT2 inhibitors on the progression of PWV remains limited [8–11].

This study evaluated the effects of tofogliflozin, an SGLT2 inhibitor that has been clinically used in Japan, on the brachial-ankle pulse wave velocity (baPWV) as one of the prespecified secondary outcomes of the using tofogliflozin for possible better intervention against atherosclerosis for type 2 diabetes patients (UTOPIA) trial [22, 23].

## Methods

### Study design

The study design, study schedule, and outcomes of the original UTOPIA trial have been described in detail previously [22, 23]. In brief, the UTOPIA trial was a prospective, randomized, open-label, multicenter, parallel-group comparative study to evaluate the efficacy of tofogliflozin in preventing the progression of atherosclerosis over a 2-year intervention period in patients with T2DM. The study period was 104 weeks following patient registration (registration period: January to November 2016). All randomized patients were followed up until the scheduled end of the study, irrespective of adherence to or discontinuation of study medication for any reason. Clinical and biochemical data were collected at 0, 26, 52, 78, and 104 weeks after randomization. Primary study outcomes were changes in mean intima–media thickness (IMT) of the common carotid artery. As one of the prespecified secondary outcomes, changes in the right and left baPWV values over the 104-week observation period were evaluated. In addition, as a post-hoc analysis, the effects of tofogliflozin on changes in the mean baPWV values (mean derived from the left- and right-side baPWV values) were evaluated.

This study was registered at the University Hospital Medical Information Network Clinical Trials Registry (UMIN-CTR), a nonprofit organization in Japan, and met the requirements of the International Committee of Medical Journal Editors (UMIN000017607).

### Study population

The eligibility criteria of the original UTOPIA trial have been previously described in detail [22]. The inclusion criteria were: (1) Japanese with T2DM and inadequate glycemic control (HbA1c ≥ 6% but < 9%), along with the inability to achieve the blood glucose level stated in the Diabetes Treatment Guidelines of 2014–2015 despite being on drugs, except SGLT2 inhibitors—with diet and physical therapy, on diet and physical therapy without being on drugs for at least 12 weeks, or on SGLT2 inhibitors in the past but without them for at least 12 weeks before signing the consent form; (2) no changes in the antidiabetic, antithrombotic, antihypertensive medication, or a therapeutic agent for dyslipidemia management for at least 12 weeks before signing the consent form; (3) age 30 to 74 years at the time of giving consent; and (4) able to provide informed consent. Furthermore, the following exclusion criteria were applied: (1) type 1 or secondary diabetes; (2) in the perioperative period or with a serious infection or injury; (3) a history of myocardial infarction, angina, stroke, or cerebral infarction; (4) severe renal dysfunction (estimated glomerular filtration rate [eGFR] of < 30 mL/min/1.73 m^2^); (5) serious liver functional impairment; 6) moderate to severe heart failure (class 3 or worse based on the New York Heart Association Functional Classification); (7) urinary tract or genital infection; (8) pregnant, possibly pregnant, nursing, or planning to conceive a child; (9) history of hypersensitivity to the study drug; (10) present or past history of a malignant tumor (exceptions: patients not on medication for malignant tumors and no recurrence of the disease so far without recurrence risks during this study were allowed to participate); (11) prohibited to use tofogliflozin; and (12) other ineligibility determined by an investigator.

The patients were screened consecutively at 24 institutions in Japan (Additional file 1). Patients who met the above eligibility criteria were asked to participate, and those who agreed were included in the study. Measurements of baPWV were performed at 10 out of 24 institutions, and the individuals who completed baPWV measurements were subjected to this analysis.

The protocol was approved by the Osaka University Clinical Research Review Committee and the institutional review board of each participating institution in compliance with the Declaration of Helsinki and current legal regulations in Japan. Written informed consent was obtained from all patients after a complete explanation of the study.

### Randomization and study intervention

As previously reported, patient registration was performed at the administration office of the UTOPIA trial using the Internet. Once enrolled, the patients were randomly and equally assigned to either tofogliflozin treatment or conventional treatment group using drugs other than SGLT2 inhibitors [22, 23].

Treatment was continued to achieve the target value specified in the Japanese treatment guide for diabetes [24] in both groups. In the conventional treatment group, either the current therapy dosage was increased or a concomitant oral glucose-lowering drug (excluding any other SGLT2 inhibitor) was added 12 weeks after randomization. In the tofogliflozin group, 20 mg of tofogliflozin once daily was started in addition to the ongoing therapy. However, the addition of an alternative antidiabetic agent (excluding another SGLT2 inhibitor) was permitted 12 weeks after randomization. In the case of hypoglycemia, the dosage of the concomitant oral glucose-lowering drug was titrated. The use of antihyperlipidemic and antihypertensive drugs was allowed during the study.

### Measurement of baPWV

The baPWV was measured using the same volume plethysmography apparatus as for ABI (BP-203RPE II form PWV/ABI, Omron Healthcare Co., Ltd., Kyoto, Japan), with patients in the supine position after at least 5 min of rest, as previously reported [25–29]. Specifically, four oscillometric cuffs, each connected to a plethysmographic sensor that determined the volume pulse from and to an oscillometric pressure sensor that measured BP, were wrapped on both the brachia and ankle; two electrocardiogram electrodes were placed on each wrist. The cuffs were simultaneously pressurized to the approximate value of the patient’s diastolic pressure such that the pulse volume waveforms could be recorded using semiconductor pressure sensors. The distance between the sampling points of baPWV was calculated automatically according to the height of the subject. The path length from the suprasternal notch to the ankle (La) was calculated as follows: La = 0.8129 × height (in cm) + 12.328. The path length from the suprasternal notch to the brachium (Lb) was calculated as follows: Lb = 0.2195 × height − 2.0734. The baPWV was calculated according to the following formula: baPWV = (La – Lb)/Tba, where Tba is the time interval between the wavefront of the brachial waveform and that of the ankle waveform [25]. Two simultaneous measurements of baPWV were recorded on the right and left sides. Investigations were conducted at the beginning of the study and 52 and 104 weeks.

### Biochemical tests

Blood samples were collected after overnight fasting. HbA1c, glucose, insulin, serum lipids, and creatinine were measured using standard techniques. Urinary albumin excretion was measured by the improved bromocresol purple method using a spot urine sample. The eGFR was calculated using the following formula: eGFR (mL/min per 1.73 m^2^) = 194 × age − 0.287 × serum creatinine − 0.1094 (× 0.739 for females) [30].

### Statistical analysis

All enrolled patients, except those without baseline baPWV measurements, were analyzed. As for baseline and follow-up variables, group comparisons were performed using Student’s *t*-test or the Wilcoxon rank-sum test for continuous variables and Fisher’s exact test or the chi-square test for categorical variables. Changes from the baseline to treatment visits were assessed with a one-sample *t*-test and Wilcoxon signed-rank test within the group.

To confirm the effect of tofogliflozin on changes in baPWV, post-hoc analyses were performed using the mixed-effects model for repeated measures, with the treatment group, time (week), interactions between treatment group and time (week), age, sex, use of insulin at baseline, and baseline baPWV as fixed effects. An unstructured covariate was used to model the covariance of within-subject variability. Sensitivity analysis assessed differences in the delta change in baPWV from the baseline between the two groups using analysis of covariance (ANCOVA) models that included the treatment group, age, sex, baseline baPWV, systolic blood pressure (SBP), and administration of antihypertensive agents.

All statistical tests were two-sided with a 5% significance level. All analyses were performed using SAS software version 9.4 (SAS Institute, Cary, NC, USA).

### Role of the funding source

The sponsor had no role in the study design, data collection, data analysis, data interpretation, or writing of the report. The corresponding author had complete access to all the data in the study and had final responsibility for the decision to submit for publication.

## Results

### Study population

**Fig. 1:**
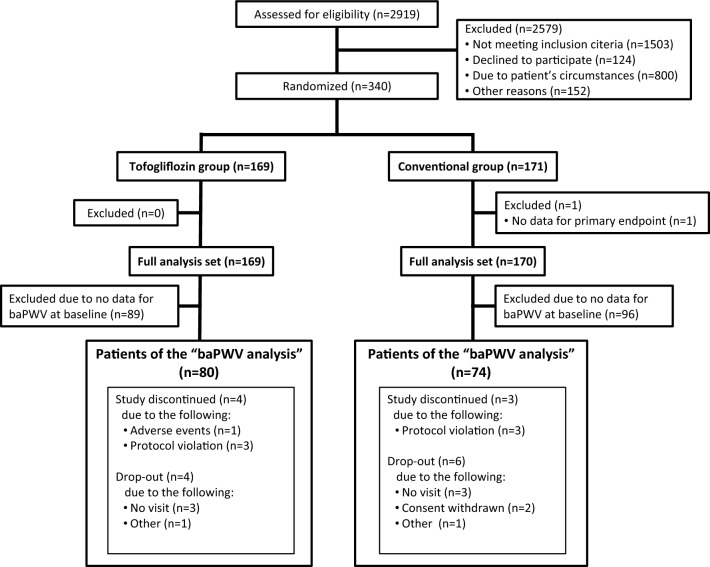
Study design flowchart

Initially, 340 patients were enrolled and randomly allocated into either the tofogliflozin treatment group (*n* = 169) or the conventional treatment group (*n* = 171). Among the study patients in the original UTOPIA trial, 154 individuals (80 belonging to the tofogliflozin group and 74 belonging to the conventional treatment group) completed baPWV measurement at baseline (Fig. 1). There were significant differences in certain variables, such as body mass index (BMI), waist circumference, SBP, total cholesterol, use of glucose-lowering agents, and use of antithrombotic agents, between those who underwent baPWV measurements and those who did not (Additional file 2).

Baseline clinical characteristics, such as sex, age, smoking habit, BMI, waist circumference, duration of diabetes, glycated hemoglobin (HbA1c), fasting blood glucose, BP, the prevalence of dyslipidemia, serum total cholesterol levels, low-density lipoprotein (LDL) cholesterol levels, high-density lipoprotein (HDL) cholesterol levels, the prevalence of diabetic retinopathy, prevalence of diabetic nephropathy, and use of glucose-lowering agents, lipid-lowering agents, and antithrombotic agents, were comparable between the tofogliflozin and conventional treatment groups, whereas serum triglyceride levels, the prevalence of hypertension, and use of antihypertensive drugs were significantly lower in the tofogliflozin treatment group than in the conventional treatment group (Table 1).

**Table 1:** Baseline clinical characteristics of patients in both treatment groups

Parameters	Tofogliflozin group (n = 80)	Conventional group (n = 74)	p value
Sex (males) (%)	48 (60.0)	48 (64.9)	0.53
Age (years)	61.5 ± 9.2	62.5 ± 9.3	0.50
Current smoking	16 (20.0)	14 (18.9)	0.87
Body mass index (kg/m^2^)	26.4 ± 5.4	26.2 ± 4.2	0.83
Waist circumference (cm)	91.2 ± 12.3 (n = 75)	91.7 ± 10.7	0.79
Duration of diabetes (years)	11.9 ± 8.2	14.1 ± 8.7	0.11
HbA1c (%)	7.5 ± 0.7	7.4 ± 0.8	0.32
Fasting blood glucose (mmol/L)	8.0 ± 1.8	7.9 ± 1.8 (n = 73)	0.65
C-peptide (ng/mL)	1.91 ± 1.11 (n = 79)	1.92 ± 0.89 (n = 73)	0.96
Hypertension	38 (47.5)	49 (66.2)	0.019
Systolic blood pressure (mmHg)	130.9 ± 14.4	132.4 ± 18.8	0.58
Diastolic blood pressure (mmHg)	79.0 ± 10.0	79.6 ± 12.0	0.70
Dyslipidemia	48 (60.0)	51 (68.9)	0.31
Total cholesterol (mmol/L)	5.01 ± 0.77 (n = 79)	5.07 ± 0.79 (n = 73)	0.63
LDL cholesterol (mmol/L)	2.88 ± 0.69	3.01 ± 0.66	0.25
HDL cholesterol (mmol/L)	1.47 ± 0.39	1.37 ± 0.27	0.09
Triglyceride (mmol/L)	1.07 [0.84, 1.61]	1.46 (1.02, 1.90)	0.011
Diabetic retinopathy	14 (17.9)	17 (23.3)	0.43
eGFR (mL/min/1.73 m^2^)	79.1 ± 19.9	82.0 ± 26.6	0.45
Urinary albumin excretion (mg/g/cre)	9.6 (5.7, 36.8) (n = 75)	18.7 (5.3, 65.9) (n = 69)	0.38
Diabetic nephropathy	24 (30.0)	27 (36.5)	0.49
Use of glucose-lowering agents	69 (86.3)	61 (82.4)	0.66
Metformin	36 (45.0)	38 (51.4)	0.52
Sulfonylurea	16 (20.0)	14 (18.9)	1.00
Glinides	4 (5.0)	4 (5.4)	1.00
Thiazolidinediones	8 (10.0)	10 (13.5)	0.62
α-Glucosidase inhibitor	14 (17.5)	15 (20.3)	0.68
DPP-4 inhibitors	36 (45.0)	34 (45.9)	1.00
GLP-1 R agonists	9 (11.3)	4 (5.4)	0.25
Insulin	12 (15.0)	16 (21.6)	0.30
Use of antihypertensive drugs	36 (45.0)	46 (62.2)	0.037
Angiotensin-converting enzyme inhibitors	1 (1.3)	4 (5.4)	0.20
Angiotensin II receptor blockers	25 (31.3)	41 (55.4)	0.003
Direct renin inhibitor	1 (1.3)	0 (0.0)	1.00
Calcium channel blocker	24 (30.0)	24 (32.4)	0.86
Diuretic drugs	4 (5.0)	6 (8.1)	0.52
α-Adrenergic receptor antagonist	1 (1.3)	0 (0.0)	1.00
β-Adrenergic receptor antagonist	3 (3.8)	2 (2.7)	1.00
Others	0 (0.0)	0 (0.0)	–
Use of lipid-lowering agents	39 (48.8)	39 (52.7)	0.63
Statins	33 (41.3)	32 (42.3)	0.87
Ezetimibe	4 (5.0)	5 (6.8)	0.74
Resins	0 (0.0)	0 (0.0)	–
Fibrates	4 (5.0)	2 (2.7)	0.68
Use of antithrombotic agents	11 (13.8)	11 (14.9)	1.00
Antiplatelet agents	10 (12.5)	9 (12.2)	1.00
Anticoagulants	1 (1.3)	2 (2.7)	0.61
Others	0 (0.0)	0 (0.0)	–
Right baPWV (cm/s)	1690.1 ± 399.2	1680.4 ± 323.6	0.87
Left baPWV (cm/s)	1696.2 ± 432.7	1672.8 ± 338.0	0.71
Mean baPWV (cm/s)	1693.2 ± 410.3	1676.6 ± 324.6	0.78

Data are presented as the number (%) of patients, mean ± standard deviation (SD) values, or median (the 25th and 75th percentiles) values

*HbA1C* glycated hemoglobin, *SD* standard deviation, *LDL* low-density lipoprotein, *HDL* high-density lipoprotein, *DPP-4* dipeptidyl peptidase, *GLP* glucagon-like peptide-1, *baPWV* brachial-ankle pulse wave velocity

Post-hoc between-group comparison of changes in clinical parameters during the treatment period was performed in 154 individuals who completed baPWV measurement at baseline. Within 104 weeks, the reductions (value at study end-value at the baseline) in HbA1c (– 0.37 ± 0.73% vs. 0.00 ± 0.68%, p = 0.002), fasting blood glucose (− 0.8 ± 2.0 mmol/L vs. 0.1 ± 2.1 mmol/L, p = 0.011), BMI (– 1.1 ± 1.1 kg/m^2^ vs. − 0.1 ± 0.8 kg/m^2^, p < 0.001), and waist circumference (− 0.9 ± 6.7 cm vs. 1.5 ± 3.9 cm, p = 0.015) were significantly higher in the tofogliflozin group than in the conventional group. There was no significant difference between the groups regarding changes in other clinical parameters including lipid profile and BP (Additional file 3). Over the course of the study, the concomitantly used antidiabetic agents were balanced between the conventional and tofogliflozin groups (Additional file 4). However, antihypertensive drugs, especially angiotensin II receptor blockers, were significantly more frequently used in the conventional group than in the tofogliflozin group during the study (Additional file 5).

### Brachial-ankle pulse wave velocity

**Fig. 2:**
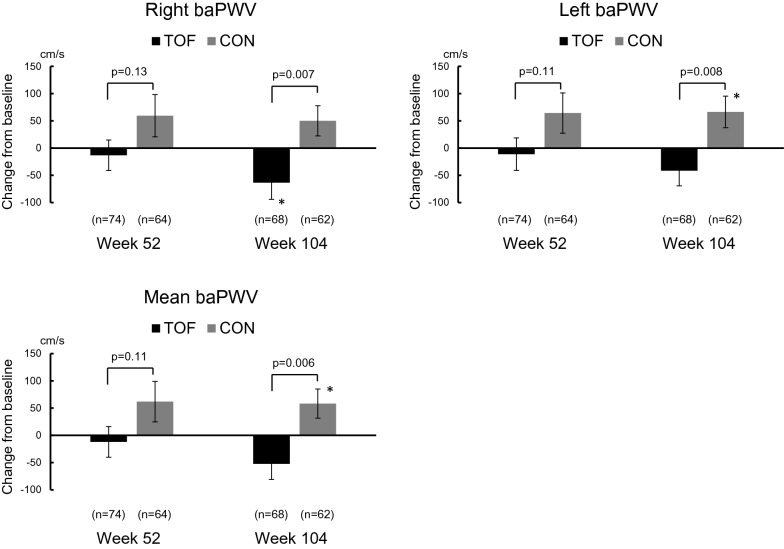
Delta change in baPWV from the baseline to weeks 52 and 104. Data are presented as the mean ± SE. Comparisons of baPWV values during treatment with those at baseline were performed using a one-sample *t*-test. *p < 0.05, ^#^*p* < 0.01, ^§^*p* < 0.001. Differences in delta change in baPWV from baseline to weeks 52 and 104 between the groups at each point (treatment effect) were analyzed using Student’s *t*-test. baPWV, brachial-ankle pulse wave velocity; TOF, tofogliflozin treatment group; CON, conventional treatment group

Figure 2 shows the change in baPWV from the baseline to weeks 52 and 104 in the tofogliflozin and conventional treatment groups. There were significant differences in delta change in the right, left, and mean baPWV from the baseline to week 104 between the treatment groups (p = 0.007, 0.008, 0.006, respectively). The progression of baPWV values was significantly attenuated in the tofogliflozin treatment group compared to the conventional treatment group.

In a mixed-effects model for repeated measures, the progression in the right, left, and mean baPWV during week 104 was significantly attenuated in the tofogliflozin group compared to that in the conventional treatment group (− 109.3 [− 184.3, − 34.3], mean change [95% CI], p = 0.005; –98.3 [− 172.6, –24.1], p = 0.010; − 104.7 [− 177.0, − 32.4], p = 0.005, respectively) (Table 2). An additional analysis, where individuals with all three measurements (baseline, week 52, and week 104) alone were included, showed similar results (Additional file 6).

**Table 2:** Effects of tofogliflozin on brachial-ankle pulse wave velocity

	Tofogliflozin group (n = 74)	Conventional group (n = 66)	Treatment effect (tofogliflozin–conventional treatment) (mean change [95% CI])	p value between groups
Right baPWV				
Change from baseline at week 52 (cm/s)	− 15.4 (38.1)	50.0 (38.3)	− 65.4 (–163.1, 32.2)	0.19
Change from baseline at week 104 (cm/s)	− 70.6 (31.3) ^*^	38.7 (30.2)	− 109.3 (–184.3, –34.3)	0.005
Left baPWV				
Change from baseline at week 52 (cm/s)	1.5 (36.9)	63.7 (37.3)	− 62.2 (–157.1, 32.7)	0.20
Change from baseline at week 104 (cm/s)	− 32.6 (30.7)	65.8 (29.8)*	− 98.3 (–172.6, –24.1)	0.010
Mean baPWV				
Change from baseline at week 52 (cm/s)	− 7.7 (36.7)	56.7 (37.0)	− 64.4 (–158.8, 29.9)	0.18
Change from baseline at week 104 (cm/s)	− 52.5 (30.1)	52.2 (29.1)	− 104.7 (–177.0, –32.4)	0.005

Data are presented as mean (SE) unless stated otherwise. Comparisons of baPWV values during treatment with those at baseline were performed using a one-sample *t*-test based on the mixed-effects model for repeated measures. *p < 0.05, ^#^p < 0.01, ^§^p < 0.001. Differences in delta change in baPWV from baseline to weeks 52 and 104 between groups at each point (treatment effect) were analyzed with the mixed-effects model for repeated measures. Treatment group, week, interactions between treatment group and week, age, sex, use of insulin at baseline, and baseline baPWV were included as fixed effects

*baPWV* brachial-ankle pulse wave velocity

Similar findings were obtained even after adjusting the mixed-effects models for traditional cardiovascular risk factors, including BMI, HbA1c, total cholesterol, HDL cholesterol, triglycerides, SBP, presence of hypertension, and smoking, and/or the administration of drugs, including hypoglycemic agents, antihypertensive agents, statins, and anti-platelets at baseline (Table 3). Moreover, the findings produced by ANCOVA models, as a sensitivity analysis, resembled those generated by the mixed-effects models (Table 4).

**Table 3:** Effects of tofogliflozin on brachial-ankle pulse wave velocity after adjusting for traditional CV risk factors and/or administration of drugs

	Model 1	Model 2	Model 3	Model 4
Right baPWV				
Week 52	− 78.3 (− 173.1, 16.6)	− 87.1 (− 186.0, 11.9)	− 90.9 (− 189.9, 8.1)	− 84.6 (− 180.6, 11.4)
Week 104	− 126.5 (− 211.0, − 42.0)^#^	− 136.9 (− 226.5, − 47.2)^#^	− 140.5 (− 230.2, − 50.7)^#^	− 134.4 (− 221.3, − 47.4)^#^
Left baPWV				
Week 52	− 77.2 (− 171.0, 16.7)	− 92.2 (− 190.0, 5.6)	− 94.6 (− 192.7, 3.4)	− 82.2 (− 178.3, 14.0)
Week 104	− 115.6 (− 196.3, − 34.9)^#^	− 132.8 (− 219.3, –46.3)^#^	− 134.6 (− 221.4, –47.8)^#^	− 121.4 (− 205.7, − 37.2)^#^
Mean baPWV				
Week 52	− 77.9 (− 170.1, 14.2)	− 89.7 (− 185.8, 6.4)	− 93.0 (− 189.2, 3.3)	− 83.6 (− 177.6, 10.3)
Week 104	− 121.1 (− 200.5, − 41.6)^#^	− 134.7 (− 219.3, –50.0)^#^	− 137.6 (− 222.5, − 52.7)^#^	− 128.0 (− 210.3, − 45.6)^#^

Treatment effect (tofogliflozin–conventional treatment) is expressed as mean change (95% CI). Differences in delta change in baPWV from the baseline at weeks 52 and 104 between the groups at each point (treatment effect) were analyzed with a mixed-effects model for repeated measures. *p < 0.05, ^#^p < 0.01, ^§^p < 0.001. Model 1: treatment group, week, interactions between treatment groups and week, body mass index, HbA1c, total cholesterol, high-density lipoprotein cholesterol, triglyceride, and systolic blood pressure were included as fixed effects. Model 2: Model 1 plus smoking, DPP-4 inhibitors, pioglitazone, angiotensin-converting enzyme/angiotensin II receptor blocker, statins, and anti-platelets were included as fixed effects. Model 3: Model 1 plus smoking, hypoglycemic agents, angiotensin-converting enzyme/angiotensin II receptor blocker, statins, and anti-platelets were included as fixed effects. Model 4: Model 1 plus hypertension, smoking, hypoglycemic agents, antihypertensive agents, statins, and anti-platelets were included as fixed effects

**Table 4:** Effects of tofogliflozin on brachial-ankle pulse wave velocity analyzed using covariance models

	Tofogliflozin treatment group	Conventional treatment group	Treatment effect (tofogliflozin–conventional treatment) (mean change [95% CI])
Right baPWV (cm/s) (mean change; SE)			
Week 52 (n = 138)	1.3 (39.9)	77.0 (38.8)	− 75.5 (− 171.1, 19.7), p = 0.12
Week 104 (n = 130)	− 67.9 (32.5)	34.7 (31.5)	− 102.7 (− 182.1, − 23.2), p = 0.012
Left baPWV (cm/s) (mean change; SE)			
Week 52 (n = 138)	− 7.1 (40.1)	76.2 (39.1)	− 83.3 (− 179.3, 12.8), p = 0.09
Week 104 (n = 130)	− 33.7 (32.2)	67.6 (31.4)	− 101.3 (− 180.3, − 22.3), p = 0.012
Mean baPWV (cm/s) (mean change; SE)			
Week 52 (n = 138)	− 4.2 (39.2)	76.9 (38.1)	− 81.2 (− 174.9, 12.6), p = 0.09
Week 104 (n = 130)	− 51.9 (31.4)	51.2 (30.5)	− 103.1 (− 180.1, − 26.2), p = 0.009

Differences in delta change in baPWV from the baseline between the two groups were analyzed using analysis of covariance models that included treatment group, age, sex, use of insulin, baseline mean baPWV, systolic blood pressure, presence of hypertension, and antihypertensive drug administration

Subgroup analysis was performed according to whether the reductions in SBP (value at week 104-value at the baseline < 0 mmHg) was observed or did not show similar results. In the subgroup without a reduction in SBP during the observation period, tofogliflozin (n = 39) significantly attenuated the progression of right, left, and mean baPWV compared to those in the conventional treatment (n = 39) (− 101.4 [− 173.0, –29.8], mean change [95%CI], p = 0.006; − 103.8 [− 182.2, − 25.5], p = 0.010; − 102.6 [− 173.6, − 31.7], p = 0.005, respectively). In the subgroup with the reduction in SBP during the observation period (tofogliflozin; n = 37, conventional treatment [n = 29], similar results were observed; however, they did not reach statistical significance (− 108.9 [− 260.2, 42.3], p = 0.15; − 99.1 [− 243.6, 45.4], p = 0.18; − 104.0 [− 246.9, 38.9], p = 0.15, respectively). The results of other subgroup analyses resembled those produced by whole-group analyses (Additional file 7).

## Discussion

Arterial stiffness is one of the earliest detectable signs of functional and structural changes in the vascular wall. baPWV, an established quantitative indicator of arterial stiffness, is linked to CV risk factors [31, 32] and is a promising predictor of future CV events independent of conventional CV risk factors [29, 33–39]. However, the effect of SGLT2 inhibitors on the progression of baPWV has not yet been completely elucidated.

To the best of our knowledge, this is the first study to demonstrate the suppression of baPWV increase by SGLT2 inhibitors in patients with T2DM without a history of CVD. Interestingly, our findings were consistent with those of several previous studies indicating the beneficial effect of SGLT2 inhibitors on the carotid-femoral PWV (cfPWV), which is currently the gold standard for measuring aortic stiffness and has been shown to predict outcomes. A single-arm, prospective clinical trial in 42 patients with T1DM revealed that 8-week treatment with empagliflozin was associated with a decline in arterial stiffness as assessed by cfPWV and carotid-radial PWV [8]. Similarly, another pilot study including 16 patients with T2DM revealed that 2-day treatment with dapagliflozin significantly improved cfPWV, systemic endothelial function, and renal resistive index [9, 10]. Furthermore, a recent, small randomized clinical trial demonstrated that canagliflozin improved cfPWV in 30 patients with T2DM and hypertension, independent of the BP effect [11]. Although the cfPWV primarily reflects aortic stiffness, baPWV is an index of arterial stiffness based on the measurements performed at peripheral sites, reflecting the wall properties not only of the aorta but also of muscle arteries. The baPWV is thus influenced by several factors modulating small- and medium-sized arteries, such as central sympathetic drive, smooth muscle tone, and spontaneous vasomotion, other than those known to influence large arteries and cfPWV. The clinical significance could be different between baPWV and cfPWV, and thus, our study adds novel findings regarding the effect of SGLT2 inhibitors on arterial stiffness.

The beneficial effects of SGLT2 inhibitors, such as tofogliflozin [12], empagliflozin [13–15], and canagliflozin [16], on arterial stiffness have been revealed in clinical studies using other indices of arterial stiffness, such as ambulatory arterial stiffness index [13], pulse pressure, mean arterial pressure, double product [16], and cardio-ankle vascular index (CAVI) [12]. However, several studies did not find beneficial effects of this class of antidiabetics on arterial stiffness [18]. Patoulias et al. recently reviewed and summarized clinical studies on this topic and concluded that evidence on the effect of SGLT2 inhibitors on arterial stiffness remains limited and controversial [19].

In addition, these clinical findings were consistent with those of an experimental study on animal models of arteriosclerosis that showed that an SGLT2 inhibitor suppressed arteriosclerosis [40–43]. Furthermore, our findings were consistent with the results of several previous studies that indicated the suppression of CV events by SGLT2 treatment in patients with high-risk T2DM [3, 4].

Interestingly, as we recently reported in a previous study, there was no significant difference in carotid IMT between the tofogliflozin and conventional treatment groups in the UTOPIA trial [23]. Several possible reasons explain the different outcomes between the observed changes in the carotid IMT (the primary outcome) and that of baPWV (a secondary outcome). One possible explanation is that the UTOPIA trial was not powered to detect significant differences between the treatment groups when evaluating IMT. Furthermore, it could be possible that the inhibition of IMT progression following tofogliflozin treatment might have been masked by the analogous effects of other drugs such as metformin, dipeptidyl peptidase (DPP)-4 inhibitors, antihypertensive drugs, and lipid-lowering agents [23]. Another possible explanation is that tofogliflozin could have different effects on IMT and baPWV. Both IMT and baPWV are indicators of atherosclerosis; however, it is believed that they reflect different aspects of atherosclerosis. Generally, atherosclerotic changes occur in the large arteries (including the carotid arteries), whereas CIMT primarily reflects the degree of atherosclerosis, particularly structural changes, developed in the large arteries [44]. In contrast, baPWV reflects a decrease in the elasticity of the arterial wall due to functional as well as structural arteriosclerotic changes that occur in the area extending from the aorta to the arteries of the extremities [20, 21]. Such a decrease in the elasticity of the arterial wall is not solely associated with atherosclerotic changes. Loss of elasticity of the arterial wall increases the vascular wall stress, resulting in atherosclerosis, an increase in the left ventricular afterload, left ventricular diastolic dysfunction, and coronary perfusion disorder. In asymptomatic patients with CV risk factors, deterioration in arterial stiffness was associated with hospitalization for new–onset heart failure [7]. Based on this, baPWV may reflect not only atherosclerotic changes but also risks of an extensive range of cardiovascular disorders including cardiac dysfunction. Therefore, changes in the baPWV, rather than in the carotid IMT, might be a better indicator of the whole CV system.

Although the beneficial effect of tofogliflozin on baPWV progression was independent of the conventional risk factors assessed at baseline, improvements in multiple CV risk factors by tofogliflozin could have inhibited the progression of arterial stiffness. Arterial stiffness reportedly improved after an intervention of classic CV risk factors (diabetes, obesity, hypertension, and hyperlipidemia). SGLT2 inhibitors have a favorable effect on these risk factors, such as lowering blood glucose levels and blood pressure as well as improving obesity and lipid profile [1, 2]. Moreover, the original UTOPIA trial showed a significant decrease in HbA1c, blood glucose levels, BMI, abdominal circumference, and SBP. Furthermore, high-density lipoprotein (HDL) cholesterol levels significantly increased [23]. Similar results were observed in this study, where patients with baPWV data at baseline were subjected (Additional file 3). Thus, improvements in the classic risk factors via an intervention could inhibit the progression of arterial stiffness.

It is believed that BP is a strong contributing factor to PWV [45]. To assess the effect of BP, we assigned patients to two groups based on their SBP during the observation period (i.e., decreased BP and unchanged/increased BP groups) for subgroup analysis. Interestingly, a positive effect of tofogliflozin on baPWV was observed in the group where patients’ SBP remained unchanged or increased during the observation period. In other words, such a favorable effect cannot be explained by the decrease in BP alone.

The major pathophysiologic mechanisms underlying increased arterial stiffness in DM include hyperglycemia, insulin resistance, the formation of advanced glycation end products (AGEs), enhanced oxidative stress, chronic inflammation, and increased activity of the renin–angiotensin–aldosterone system [43]. In addition, our previous study indicated that elevated levels of plasma indoxyl sulfate, a uremic toxin, was related to increased baPWV [46]. Although it has been demonstrated that SGLT2 inhibition ameliorates insulin resistance, decreases the expression of AGEs, and suppresses oxidative stress and inflammatory response [19], these aspects were not elucidated in the present study.

### Limitations

This study had several limitations. First, the number of study subjects was small and the change in baPWV over time was a secondary outcome in the UTOPIA trial. In addition, not all patients who were enrolled in the UTOPIA trial underwent baPWV evaluation. There were significant differences in certain variables at baseline between those who underwent baPWV measurements and those who did not (Additional file 2), which was not irrelevant to bias. Therefore, results should be interpreted with caution and further investigation in a large-scale study that uses changes in baPWV over time as the primary outcome is required.

Second, rates of complication with hypertension and the use of antihypertensive drugs were lower in the tofogliflozin than in the normal treatment group at baseline (Additional file 5). Such a tendency was observed throughout the study period and may have affected the study results. However, multivariate analysis after adjusting for concomitant drugs and several CV risk factors, including BP, confirmed a significant association between tofogliflozin treatment and the inhibition of baPWV progression (Tables 3 and 4).

Third, several studies have demonstrated baPWV as an independent risk factor for CVDs. In addition, various studies have been conducted for antihypertensive [47, 48] and antidiabetic agents [49, 50] using changes in baPWV over time as an outcome. However, there is insufficient evidence about whether inhibiting the increase in baPWV reflects a lowered risk of the onset of CVDs. Hence, it would be too early to conclude that tofogliflozin reduces the risk of onset of CVDs in patients with T2DM without a history of CVD.

Finally, the patients in this study were Japanese with T2DM, a cohort with relatively low CV risks. Therefore, it would be premature to generalize our findings to other racial or ethnic groups.

## Conclusion

This study showed that tofogliflozin significantly inhibited the increase in baPWV in patients with T2DM without a history of CVD, and suggested that tofogliflozin suppressed the progression of arterial stiffness.

## Supplementary Information

**Additional file 1:** UTOPIA trial site investigators.

**Additional file 2:** **Table S1.** Clinical characteristics of patients with and without brachial-ankle pulse wave velocity data.

**Additional file 3:** **Table S2.** Between-group comparison of changes in clinical parameters during the treatment period.

**Additional file 4:** **Table S3.** Changes in concomitantly used anti-diabetic agents.

**Additional file 5:** **Table S4.** Changes in concomitantly used cardiovascular medications.

**Additional file 6:** **Table S5.** Effects of tofogliflozin on brachial-ankle pulse wave velocity in individuals with all three measurements (baseline, week 52, and week 104).

**Additional file 7:** **Table S6.** Effects of tofogliflozin on brachial-ankle pulse wave velocity change in subgroups.

## Data Availability

The datasets generated and/or analyzed during our study are available from the corresponding author upon reasonable request.

## Abbreviations

ANCOVA: Analysis of covariance
BMI: Body mass index
eGFR: Estimated glomerular filtration rate
IMT: Intima–media thickness
SGLT2: Sodium-glucose cotransporter 2
UTOPIA: Using Tofogliflozin for possible better intervention against atherosclerosis for type 2 diabetes patients
