# Supplementary material for: Effect of tofogliflozin on arterial stiffness in patients with type 2 diabetes: prespecified sub-analysis of the prospective, randomized, open-label, parallel-group comparative UTOPIA trial
Source: Cardiovasc Diabetol. 2021 Jan 4;20:4. doi: 10.1186/s12933-020-01206-1 (PMC7784389; doi:10.1186/s12933-020-01206-1)
Supplement: Supplementary file 1 — Additional file 1: UTOPIA trial site investigators. [file 12933_2020_1206_MOESM1_ESM.docx]

**Additional file 1: UTOPIA trial site investigators (listed in alphabetical order):**

*Hayashi Clinic:* I. Hayashi

*Ikeda Municipal Hospital:* M. Tsugawa

*Jiyugaoka Medical Clinic:* H. Yokoyama

*Juntendo Tokyo Koto Geriatric Medical Center:* H. Yoshii

*Juntendo University Graduate School of Medicine:* K. Komiyama, T. Mita, T. Shimizu

*Kansai Rosai Hospital:* T. Yamamoto

*Kanda Naika Clinic:* S. Kawashima

*Kawasaki Hospital:* T. Nakamura

*Kawasaki Medical School:* S. Kamei, T. Kinoshita, M. Shimoda

*Kitasenri Maeda Clinic:* K. Maeda

*Kosugi Medical Clinic:* K. Kosugi

*Misaki Naika Clinic:* H. Yoshii

*NakaKinen Clinic:* H. Ishida, T. Osonoi, M. Saito, A. Tamazawa

*Nissay Hospita:* S. Sumitani

*Osaka General Medical Center:* N. Fujiki, Y. Fujita, S. Shimizu, Y. Umayahara

*National Hospital Organization Osaka National Hospital:* K. Kato

*Osaka Police Hospital:* Y. Irie, R. Kataoka, T. Yasuda

*Osaka Rosai Hospital:* Y. Kiyohara, M. Ohashi, K. Ryomoto, Y. Takahi

*Osaka University Graduate School of Medicine:* Y. Fujishima, Y. Fujita, A. Fukuhara, K. Fukui, Y. Hosokawa, A. Imagawa, H. Iwahashi, K. Mukai, N. Katakami, T. Katsura, D. Kawamori, T. Kimura, S. Kobayashi, J. Kozawa, F. Kubo, N. Maeda, T. Matsuoka, K. Miyashita, S. Nakata, H. Ninomiya, H. Nishizawa, Y. Okuno, M. Otsuki, F. Sakamoto, S. Sasaki, I. Sato, N. Shimo, I. Shimomura, M. Takahara, T. Takano, A. Tokunaga, S. Uno, M. Yamaoka, S. Yoneda

*Otoshi Medical Clinic:* K. Ohtoshi

*Shiraiwa Medical Clinic:* T. Shiraiwa

*University of Occupational and Environmental Health, Japan:* M. Hajime, K. Koikawa, F. Kuno, A. Kurozumi, K. Matsushita, M. Narisawa, K. Tanaka, K. Sugai, Y. Okada, K. Torimoto
