# Supplementary material for: Effect of tofogliflozin on arterial stiffness in patients with type 2 diabetes: prespecified sub-analysis of the prospective, randomized, open-label, parallel-group comparative UTOPIA trial
Source: Cardiovasc Diabetol. 2021 Jan 4;20:4. doi: 10.1186/s12933-020-01206-1 (PMC7784389; doi:10.1186/s12933-020-01206-1)
Supplement: Supplementary file 2 — Additional file 2: Table S1. Clinical characteristics of patients with and without brachial-ankle pulse wave velocity data. [file 12933_2020_1206_MOESM2_ESM.docx]

**Additional file 2: Table S1. Clinical characteristics of patients with and without brachial–ankle pulse wave velocity data**

| Parameters | Patients with baPWV data (n = 154) | Patients without baPWV data (n = 185) | p value |
| --- | --- | --- | --- |
| Sex (males) (%) | 96 (62.3) | 102 (55.1) | 0.18 |
| Age (years) | 62.0 ± 9.2 | 60.4 ± 9.7 | 0.13 |
| Current smoking | 30 (19.5) | 37 (20.1) | 0.45 |
| Body mass index (kg/m^2^) | 26.3 ± 4.9 | 27.6 ± 5.4 | 0.017 |
| Waist circumference (cm) | 91.5 ± 11.5 (n = 149) | 95.2 ± 12.6 (n = 154) | 0.007 |
| Duration of diabetes (years) | 12.9 ± 8.5 (n = 150) | 11.8 ± 8.2 (n = 169) | 0.21 |
| HbA1c (%) | 7.4 ± 0.8 | 7.3 ± 0.7 | 0.24 |
| Fasting blood glucose (mmol/L) | 7.9 ± 1.8 (n = 153) | 7.7 ± 1.7 (n = 182) | 0.32 |
| C-peptide (ng/mL) | 1.9 ± 1.0 (n = 152) | 2.0 ± 1.3 (n = 182) | 0.39 |
| Hypertension | 87 (56.5) | 106 (57.3) | 0.91 |
| Systolic blood pressure (mmHg) | 131.6 ± 16.7 | 135.7 ± 15.1 (n = 176) | 0.021 |
| Diastolic blood pressure (mmHg) | 79.3 ± 11.0 | 77.6 ± 10.0 (n = 176) | 0.14 |
| Dyslipidemia | 99 (64.3) | 130 (70.3) | 0.25 |
| Total cholesterol (mmol/L) | 5.0 ± 0.8 (n = 152) | 4.9 ± 0.8 (n = 176) | 0.035 |
| LDL cholesterol (mmol/L) | 2.9 ± 0.7 | 2.8 ± 0.7 (n = 183) | 0.15 |
| HDL cholesterol (mmol/L) | 1.4 ± 0.3 | 1.4 ± 0.3 | 0.19 |
| Triglyceride (mmol/L) | 1.2 (0.9, 1.8) | 1.4 (1.0, 1.9) (n = 182) | 0.11 |
| Diabetic retinopathy | 31 (20.5) | 30 (16.2) | 0.31 |
| Diabetic nephropathy | 51 (33.1) | 50 (27.0) | 0.23 |
| Use of glucose-lowering agents | 130 (84.4) | 175 (94.6) | 0.003 |
| Metformin | 74 (48.1) | 117 (63.2) | 0.006 |
| Sulfonylurea | 30 (19.5) | 51 (27.6) | 0.10 |
| Glinides | 8 (5.2) | 12 (6.5) | 0.65 |
| Thiazolidinediones | 18 (11.7) | 23 (12.4) | 0.87 |
| α-Glucosidase inhibitor | 29 (18.8) | 20 (10.8) | 0.044 |
| DPP-4 inhibitors | 70 (45.5) | 100 (54.1) | 0.13 |
| GLP-1 R agonists | 13 (8.4) | 22 (11.9) | 0.37 |
| Insulin | 28 (18.2) | 44 (23.8) | 0.23 |
| Use of antihypertensive drugs | 82 (53.2) | 92 (49.7) | 0.59 |
| Angiotensin-converting enzyme inhibitors | 5 (3.2) | 3 (1.6) | 0.48 |
| Angiotensin II receptor blockers | 66 (42.9) | 80 (43.2) | 1.00 |
| Direct renin inhibitor | 1 (0.6) | 1 (0.5) | 1.00 |
| Calcium channel blocker | 48 (31.2) | 53 (28.6) | 0.63 |
| Diuretic drugs | 10 (6.5) | 12 (6.5) | 1.00 |
| α-Adrenergic receptor antagonist | 1 (0.6) | 1 (0.5) | 1.00 |
| β-Adrenergic receptor antagonist | 5 (3.2) | 1 (0.5) | 0.10 |
| Others | 0 (0.0) | 0 (0.0) | – |
| Use of lipid-lowering agents | 78 (50.6) | 103 (55.7) | 0.38 |
| Statins | 65 (42.2) | 91 (49.2) | 0.23 |
| Ezetimibe | 9 (5.8) | 12 (6.5) | 1.00 |
| Resins | 0 (0.0) | 1 (0.5) | 1.00 |
| Fibrates | 6 (3.9) | 8 (4.3) | 1.00 |
| Use of antithrombotic agents | 22 (14.3) | 10 (5.4) | 0.008 |
| Antiplatelet agents | 19 (12.3) | 7 (3.8) | 0.004 |
| Anticoagulants | 3 (1.9) | 3 (1.6) | 1.00 |
| Others | 0 (0.0) | 0 (0.0) | – |

Data are presented as number (%) of patients or mean ± SD values. HbA1C: glycated hemoglobin; SD: standard deviation; LDL: low-density lipoprotein; HDL: high-density lipoprotein; DPP-4: dipeptidyl peptidase; GLP: glucagon-like peptide-1.
