# Supplementary material for: Effect of tofogliflozin on arterial stiffness in patients with type 2 diabetes: prespecified sub-analysis of the prospective, randomized, open-label, parallel-group comparative UTOPIA trial
Source: Cardiovasc Diabetol. 2021 Jan 4;20:4. doi: 10.1186/s12933-020-01206-1 (PMC7784389; doi:10.1186/s12933-020-01206-1)
Supplement: Supplementary file 3 — Additional file 3: Table S2. Between-group comparison of changes in clinical parameters during the treatment period. [file 12933_2020_1206_MOESM3_ESM.docx]

**Additional file 3: Table S2.**

**B****etween-group comparison of changes in clinical parameters during the treatment period**

| Parameters | Tofogliflozin group | Conventional group | p value |
| --- | --- | --- | --- |
| Body mass index at baseline (kg/m^2^) | 26.4 ± 5.4 (n = 80) | 26.2 ± 4.2 (n = 74) | 0.83 |
| Week 26 (change from baseline) | –0.7 ± 0.8 (n = 78)§ | 0.0 ± 0.8 (n = 72) | < 0.001 |
| Week 52 (change from baseline) | –0.9 ± 1.2 (n = 79)§ | 0.0 ± 0.9 (n = 70) | < 0.001 |
| Week 78 (change from baseline) | –0.9 ± 1.2 (n = 77)§ | 0.1 ± 0.9 (n = 68) | < 0.001 |
| Week 104 (change from baseline) | –1.1 ± 1.1 (n = 77)§ | –0.1 ± 0.8 (n = 68) | < 0.001 |
| Waist circumference at baseline (cm) | 91.2 ± 12.3 (n = 75) | 91.7 ± 10.7 (n = 74) | 0.79 |
| Week 26 (change from baseline) | –2.1 ± 6.5 (n = 65)^*^ | 1.2 ± 4.6 (n = 65)^*^ | 0.001 |
| Week 52 (change from baseline) | –0.6 ± 7.3 (n = 66) | 1.8 ± 4.9 (n = 69)# | 0.026 |
| Week 78 (change from baseline) | –1.1 ± 7.8 (n = 63) | 1.6 ± 4.8 (n = 66) # | 0.021 |
| Week 104 (change from baseline) | –0.9 ± 6.7 (n = 66) | 1.5 ± 3.9 (n = 66) # | 0.015 |
| HbA1c at baseline (%) | 7.5 ± 0.7 (n = 80) | 7.4 ± 0.8 (n = 74) | 0.32 |
| HbA1c at baseline (mmol/mol) | 58.2 ± 8.1 (n = 80) | 56.8 ± 8.7 (n = 74) | 0.32 |
| Week 26 (change from baseline) | –0.38 ± 0.60 (n = 79)§ | 0.02 ± 0.52 (n = 72) | < 0.001 |
| Week 52 (change from baseline) | –0.33 ± 0.70 (n = 79)§ | 0.00 ± 0.53 (n = 70) | 0.001 |
| Week 78 (change from baseline) | –0.36 ± 0.78 (n = 77)§ | –0.08 ± 0.51 (n = 69) | 0.012 |
| Week 104 (change from baseline) | –0.37 ± 0.73 (n = 77)§ | 0.00 ± 0.68 (n = 67) | 0.002 |
| Fasting blood glucose at baseline (mmol/L) | 8.0 ± 1.8 (n = 80) | 7.9 ± 1.8 (n = 73) | 0.65 |
| Week 26 (change from baseline) | –0.9 ± 1.7 (n = 77)§ | 0.3 ± 2.2 (n = 68) | < 0.001 |
| Week 52 (change from baseline) | –1.0 ± 1.8 (n = 73)§ | 0.0 ± 1.9 (n = 66) | 0.002 |
| Week 78 (change from baseline) | –0.6 ± 1.8 (n = 75)# | –0.1 ± 2.1 (n = 66) | 0.15 |
| Week 104 (change from baseline) | –0.8 ± 2.0 (n = 76)§ | 0.1 ± 2.1 (n = 68) | 0.011 |
| C-peptide at baseline (ng/mL) | 1.91 ± 1.11 (n = 79) | 1.92 ± 0.89 (n = 73) | 0.96 |
| Week 52 (change from baseline) | –0.29 ± 0.92 (n= 74)^*^ | –0.21 ± 0.97 (n = 69) | 0.64 |
| Week 104 (change from baseline) | –0.22 ± 0.83 (n= 75)^*^ | –0.04 ± 0.92 (n = 67) | 0.23 |
| Total cholesterol at baseline (mmol/L) | 5.01 ± 0.77 (n = 79) | 5.07 ± 0.79 (n = 73) | 0.63 |
| Week 26 (change from baseline) | –0.02 ± 0.51 (n = 78) | –0.01 ± 0.55 (n = 69) | 0.93 |
| Week 52 (change from baseline) | 0.03 ± 0.61 (n = 77) | 0.00 ± 0.52 (n = 70) | 0.77 |
| Week 78 (change from baseline) | 0.04 ± 0.55 (n = 74) | 0.00 ± 0.65 (n = 69) | 0.68 |
| Week 104 (change from baseline) | 0.01 ± 0.60 (n = 75) | –0.03 ± 0.77 (n = 68) | 0.79 |
| LDL cholesterol at baseline (mmol/L) | 2.88 ± 0.69 (n = 80) | 3.01 ± 0.66 (n = 74) | 0.25 |
| Week 26 (change from baseline) | –0.07 ± 0.52 (n = 79) | –0.04 ± 0.46 (n = 71) | 0.73 |
| Week 52 (change from baseline) | –0.02 ± 0.50 (n = 79) | 0.00 ± 0.41 (n = 70) | 0.72 |
| Week 78 (change from baseline) | 0.02 ± 0.48 (n = 77) | –0.03 ± 0.60 (n = 68) | 0.59 |
| Week 104 (change from baseline) | –0.05 ± 0.58 (n = 77) | –0.08 ± 0.60 (n = 68) | 0.77 |
| HDL cholesterol at baseline (mmol/L) | 1.47 ± 0.39 (n = 80) | 1.37 ± 0.27 (n = 74) | 0.09 |
| Week 26 (change from baseline) | 0.04 ± 0.19 (n = 79) | –0.03 ± 0.16 (n = 71) | 0.031 |
| Week 52 (change from baseline) | 0.05 ± 0.17 (n = 79)^*^ | 0.00 ± 0.16 (n = 70) | 0.10 |
| Week 78 (change from baseline) | 0.06 ± 0.18 (n = 77)# | 0.01 ± 0.20 (n = 69) | 0.10 |
| Week 104 (change from baseline) | 0.06 ± 0.19 (n = 77)# | 0.02 ± 0.19 (n = 68)^*^ | 0.29 |
| Triglyceride at baseline (mmol/L) | 1.07 (0.84, 1.61) (n = 80) | 1.46 (1.02, 1.90) (n = 74) | 0.011 |
| Week 26 (change from baseline) | –0.04 (–0.34, 0.24) (n = 77) | 0.03 (–0.26, 0.36) (n = 67) | 0.69 |
| Week 52 (change from baseline) | –0.03 (–0.25, 0.23) (n = 73) | –0.03 (–0.37, 0.20) (n = 66) | 0.14 |
| Week 78 (change from baseline) | –0.01 (–0.30, 0.23) (n = 75) | –0.01 (–0.34, 0.29) (n = 66) | 0.79 |
| Week 104 (change from baseline) | –0.05 (–0.36, 0.25) (n = 76) | –0.03 (–0.35, 0.30) (n = 67) | 0.49 |
| Systolic blood pressure (mmHg) | 130.9 ± 14.4 (n = 80) | 132.4 ± 18.8 (n = 74) | 0.58 |
| Week 26 (change from baseline) | –3.7 ± 14.9 (n = 78)^*^ | –1.0 ± 16.0 (n = 72) | 0.29 |
| Week 52 (change from baseline) | –4.8 ± 11.8 (n = 79)§ | –3.0 ± 16.6 (n = 70) | 0.45 |
| Week 78 (change from baseline) | –4.2 ± 14.4 (n = 77)^*^ | –0.1 ± 17.3 (n = 69) | 0.12 |
| Week 104 (change from baseline) | –3.3 ± 14.1 (n = 76)^*^ | 0.7 ± 16.8 (n = 68) | 0.12 |
| Diastolic blood pressure (mmHg) | 79.0 ± 10.0 (n = 80) | 79.6 ± 12.0 (n = 74) | 0.70 |
| Week 26 (change from baseline) | –2.5 ± 8.1 (n = 78)^#^ | –0.4 ± 9.4 (n = 72) | 0.15 |
| Week 52 (change from baseline) | –4.7 ± 9.5 (n = 79)^§^ | –2.4 ± 10.3 (n = 70) | 0.16 |
| Week 78 (change from baseline) | –2.9 ± 9.8 (n = 77)^*^ | –1.6 ± 9.7 (n = 69) | 0.42 |
| Week 104 (change from baseline) | –4.6 ± 9.6 (n = 76)^§^ | –2.0 ± 9.4 (n = 68) | 0.11 |
| eGFR (mL/min/1.73 m^2^) | 79.1 ± 19.9 (n = 80) | 82.0 ± 26.6 (n = 74) | 0.45 |
| Week 26 (change from baseline) | –2.5 ± 7.9 (n = 79)^#^ | –3.8 ± 9.9 (n = 71)# | 0.39 |
| Week 52 (change from baseline) | –3.0 ± 8.8 (n = 79)^#^ | –3.7 ± 9.3 (n = 70)^#^ | 0.63 |
| Week 78 (change from baseline) | –4.6 ± 8.8 (n = 77)^§^ | –5.5 ± 10.0 (n = 69)^§^ | 0.54 |
| Week 104 (change from baseline) | –3.3 ± 10.5 (n = 77)^#^ | –6.6 ± 10.9 (n = 68)^§^ | 0.07 |
| UAE at baseline (mg/g/cre) | 9.6 (5.7, 36.8) (n = 75) | 18.7 (5.3, 65.9) (n = 69) | 0.38 |
| Week 26 (change from baseline) | –0.1 (–6.0, 6.7) (n = 67) | 0.4 (–8.2, 11.4) (n = 63) | 0.55 |
| Week 52 (change from baseline) | –1.7 (–8.8, 2.0) (n = 72)^*^ | 0.7 (–5.7, 12.4) (n = 65) | 0.025 |
| Week 78 (change from baseline) | –1.9 (–8.0, 4.3) (n = 68) | 0.8 (–6.8, 10.1) (n = 64) | 0.05 |
| Week 104 (change from baseline) | 0.1 (–5.6, 10.1) (n = 71) | 2.3 (–2.4, 18.4)^*^ | 0.11 |
| hsCRP at baseline (ng/mL) | 545.0 (244.0, 1170.0) (n = 79) | 575.5 (295.0, 1080.0) (n = 74) | 0.68 |
| Week 52 (change from baseline) | –31.5 (–230.0, 207.5) (n = 76) | 10.0 (–227.0, 423.0) (n = 69) | 0.33 |
| Week 104 (change from baseline) | –6.0 (–395.5, 197.0) (n = 76) | 1.0 (–271.0, 227.0) (n = 67) | 0.70 |
| Adiponectin at baseline (μg/mL) | 7.80 (5.90, 10.50) (n = 79) | 7.15 (5.00, 11.20) (n = 74) | 0.86 |
| Week 52 (change from baseline) | 0.65 (0.00, 1.30) (n = 76)^§^ | 0.30 (–0.40, 0.80) (n = 69)^*^ | 0.038 |
| Week 104 (change from baseline) | 0.75 (–0.30, 1.75) (n = 76)^§^ | 0.00 (–0.50, 1.00) (n = 67) | 0.033 |
| NT-proBNP at baseline (pg/mL) | 31.0 (17.0, 59.0) (n = 79) | 35.5 (17.0, 59.0) (n = 74) | 0.017 |
| Week 52 (change from baseline) | –1.0 (–14.0, 12.0) (n = 76) | –4.0 (–14.0, 9.0) (n = 69) | 0.63 |
| Week 104 (change from baseline) | 2.0 (9.0, 16.5) (n = 76) | 0.0 (–10.0, 11.0) (n = 67) | 0.68 |

Data are presented as mean ± SD or median ( 25^th^ and 75^th^ percentiles) values. Differences in parameters between groups at baseline were analyzed using Student’s *t*-test or the Wilcoxon rank-sum test. Differences in parameters from baseline to weeks 26, 52, 78, and 104 within each group were analyzed using a one-sample *t*-test or the Wilcoxon signed-rank test. Differences in parameters from baseline to weeks 26, 52, 78, and 104 between groups were analyzed using Student’s *t*-test or the Wilcoxon rank-sum test. ^*^p < 0.05, ^#^ p < 0.01, ^§^ p < 0.001.

eGFR, estimated glomerular filtration rate; SD: standard deviation; UAE, urinary albumin excretion; hs-CRP, high-sensitivity C-reactive protein; NT-proBNP, N-terminal–pro-brain natriuretic peptide; HbA1C: glycated hemoglobin; LDL: low-density lipoprotein; HDL: high-density lipoprotein.
