# Supplementary material for: Effect of tofogliflozin on arterial stiffness in patients with type 2 diabetes: prespecified sub-analysis of the prospective, randomized, open-label, parallel-group comparative UTOPIA trial
Source: Cardiovasc Diabetol. 2021 Jan 4;20:4. doi: 10.1186/s12933-020-01206-1 (PMC7784389; doi:10.1186/s12933-020-01206-1)
Supplement: Supplementary file 4 — Additional file 4: Table S3. Changes in concomitantly used anti-diabetic agents. [file 12933_2020_1206_MOESM4_ESM.docx]

**Additional file 4: Table S3. Changes in** **concomitantly used anti-diabetic agents**

| **Parameters** | **Tofogliflozin treatment group** | **Conventional treatment group** | p **value** |
| --- | --- | --- | --- |
| Any concomitantly used anti-diabetic agent^*^ |  |  |  |
| Baseline | 69 (86.3) | 61 (82.4) | 0.66 |
| Week 26 | 67 (84.8) | 62 (83.8) | 1.00 |
| Week 52 | 67 (84.8) | 59 (83.1) | 0.83 |
| Week 78 | 66 (84.6) | 60 (84.5) | 1.00 |
| Week 104 | 64 (83.1) | 57 (83.8) | 1.00 |
| Metformin |  |  |  |
| Baseline | 36 (45.0) | 38 (51.4) | 0.52 |
| Week 26 | 36 (45.6) | 38 (51.4) | 0.52 |
| Week 52 | 36 (45.6) | 36 (50.7) | 0.62 |
| Week 78 | 36 (46.2) | 36 (50.7) | 0.62 |
| Week 104 | 35 (45.5) | 35 (51.5) | 0.51 |
| Sulfonylurea |  |  |  |
| Baseline | 16 (20.0) | 14 (18.9) | 1.00 |
| Week 26 | 13 (16.5) | 14 (18.9) | 0.83 |
| Week 52 | 14 (17.7) | 14 (19.7) | 0.83 |
| Week 78 | 14 (17.9) | 14 (19.7) | 0.84 |
| Week 104 | 12 (15.6) | 14 (20.6) | 0.52 |
| Glinides |  |  |  |
| Baseline | 4 (5.0) | 4 (5.4) | 1.00 |
| Week 26 | 3 (3.8) | 4 (5.4) | 0.71 |
| Week 52 | 3 (3.8) | 3 (4.2) | 1.00 |
| Week 78 | 3 (3.8) | 3 (4.2) | 1.00 |
| Week 104 | 4 (5.2) | 2 (2.9) | 0.68 |
| Thiazolidinediones |  |  |  |
| Baseline | 8 (10.0) | 10 (13.5) | 0.62 |
| Week 26 | 7 (8.9) | 11 (14.9) | 0.32 |
| Week 52 | 8 (10.1) | 11 (15.5) | 0.34 |
| Week 78 | 8 (10.3) | 10 (14.1) | 0.62 |
| Week 104 | 8 (10.4) | 9 (13.2) | 0.62 |
| α-glucosidase inhibitors |  |  |  |
| Baseline | 14 (17.5) | 15 (20.3) | 0.68 |
| Week 26 | 14 (17.7) | 16 (21.6) | 0.68 |
| Week 52 | 14 (17.7) | 13 (18.3) | 1.00 |
| Week 78 | 14 (17.9) | 13 (18.3) | 1.00 |
| Week 104 | 13 (16.9) | 13 (19.1) | 0.83 |
| DPP-4 inhibitors |  |  |  |
| Baseline | 36 (45.0) | 34 (45.9) | 1.00 |
| Week 26 | 34 (43.0) | 35 (47.3) | 0.63 |
| Week 52 | 35 (44.3) | 32 (45.1) | 1.00 |
| Week 78 | 35 (44.9) | 32 (45.1) | 1.00 |
| Week 104 | 34 (44.2) | 32 (47.1) | 0.74 |
| GLP-1 R agonists |  |  |  |
| Baseline | 9 (11.3) | 4 (5.4) | 0.25 |
| Week 26 | 9 (11.4) | 4 (5.4) | 0.25 |
| Week 52 | 9 (11.4) | 4 (5.6) | 0.25 |
| Week 78 | 9 (11.5) | 4 (5.6) | 0.25 |
| Week 104 | 8 (10.4) | 3 (4.4) | 0.22 |
| Insulins |  |  |  |
| Baseline | 12 (15.0) | 16 (21.6) | 0.30 |
| Week 26 | 11 (13.9) | 16 (21.6) | 0.29 |
| Week 52 | 11 (13.9) | 18 (25.4) | 0.10 |
| Week 78 | 10 (12.8) | 17 (23.9) | 0.09 |
| Week 104 | 10 (13.0) | 15 (22.1) | 0.19 |

Data are presented as number (%) of patients. The two treatment groups were compared using Fisher’s exact test. ^*^Administration of tofogliflozin in the tofogliflozin treatment group was not counted as a concomitant anti-diabetic agent. DPP-4: dipeptidyl peptidase; GLP: glucagon-like peptide-1.
