# Supplementary material for: Effect of tofogliflozin on arterial stiffness in patients with type 2 diabetes: prespecified sub-analysis of the prospective, randomized, open-label, parallel-group comparative UTOPIA trial
Source: Cardiovasc Diabetol. 2021 Jan 4;20:4. doi: 10.1186/s12933-020-01206-1 (PMC7784389; doi:10.1186/s12933-020-01206-1)
Supplement: Supplementary file 5 — Additional file 5: Table S4. Changes in concomitantly used cardiovascular medications. [file 12933_2020_1206_MOESM5_ESM.docx]

**Additional file 5:** **Table S4. Changes in concomitantly used cardiovascular medications**

|  | **Tofogliflozin treatment group** | **Conventional treatment group** | ***P*-value** |
| --- | --- | --- | --- |
| Antihypertensive drugs |  |  |  |
| Any antihypertensive drugs |  |  |  |
| Baseline | 36 (45.0) | 46 (62.2) | 0.037 |
| Week 26 | 35 (44.3) | 47 (63.5) | 0.023 |
| Week 52 | 35 (44.3) | 46 (64.8) | 0.014 |
| Week 78 | 33 (42.3) | 45 (63.4) | 0.014 |
| Week 104 | 33 (42.9) | 42 (61.8) | 0.030 |
| Angiotensin-converting enzyme inhibitors |  |  |  |
| Baseline | 1 (1.3) | 4 (5.4) | 0.20 |
| Week 26 | 1 (1.3) | 4 (5.4) | 0.20 |
| Week 52 | 1 (1.3) | 4 (5.6) | 0.19 |
| Week 78 | 1 (1.3) | 4 (5.6) | 0.19 |
| Week 104 | 1 (1.3) | 4 (5.9) | 0.19 |
| Angiotensin II receptor blockers |  |  |  |
| Baseline | 25 (31.3) | 41 (55.4) | 0.003 |
| Week 26 | 24 (30.4) | 42 (56.8) | 0.001 |
| Week 52 | 24 (30.4) | 40 (56.3) | 0.002 |
| Week 78 | 22 (28.2) | 40 (56.3) | <0.001 |
| Week 104 | 20 (26.0) | 37 (54.4) | <0.001 |
| Calcium channel blockers |  |  |  |
| Baseline | 24 (30.0) | 24 (32.4) | 0.86 |
| Week 26 | 23 (29.1) | 25 (33.8) | 0.60 |
| Week 52 | 23 (29.1) | 27 (38.0) | 0.30 |
| Week 78 | 22 (28.2) | 27 (38.0) | 0.22 |
| Week 104 | 23 (29.9) | 24 (35.3) | 0.59 |
| Lipid-lowering agents |  |  |  |
| Any lipid-lowering agent |  |  |  |
| Baseline | 39 (48.8) | 39 (52.7) | 0.63 |
| Week 26 | 39 (49.4) | 39 (52.7) | 0.75 |
| Week 52 | 39 (49.4) | 39 (54.9) | 0.52 |
| Week 78 | 40 (51.3) | 41 (57.7) | 0.51 |
| Week 104 | 39 (50.6) | 38 (55.9) | 0.62 |
| Statins |  |  |  |
| Baseline | 33 (41.3) | 32 (43.2) | 0.87 |
| Week 26 | 33 (41.8) | 32 (43.2) | 0.87 |
| Week 52 | 33 (41.8) | 33 (46.5) | 0.62 |
| Week 78 | 33 (42.3) | 36 (50.7) | 0.33 |
| Week 104 | 33 (42.9) | 34 (50.0) | 0.41 |
| Antithrombotic agents |  |  |  |
| Any antithrombotic agent |  |  |  |
| Baseline | 11 (13.8) | 11 (14.9) | 1.00 |
| Week 26 | 11 (13.9) | 10 (13.5) | 1.00 |
| Week 52 | 11 (13.9) | 10 (14.1) | 1.00 |
| Week 78 | 10 (12.8) | 10 (14.1) | 1.00 |
| Week 104 | 10 (13.0) | 8 (11.8) | 1.00 |
| Antiplatelet drugs |  |  |  |
| Baseline | 10 (12.5) | 9 (12.2) | 1.00 |
| Week 26 | 10 (12.7) | 8 (10.8) | 0.80 |
| Week 52 | 10 (12.7) | 8 (11.3) | 1.00 |
| Week 78 | 9 (11.5) | 8 (11.3) | 1.00 |
| Week 104 | 9 (11.7) | 7 (10.3) | 1.00 |
| Anticoagulants |  |  |  |
| Baseline | 1 (1.3) | 2 (2.7) | 0.61 |
| Week 26 | 1 (1.3) | 4 (2.7) | 0.61 |
| Week 52 | 1 (1.3) | 2 (2.8) | 0.60 |
| Week 78 | 1 (1.3) | 2 (2.8) | 0.61 |
| Week 104 | 1 (1.3) | 1 (1.5) | 1.00 |

Data are presented as number (%) or mean ± SD. The two treatment groups were compared using Fisher’s exact test. SD: standard deviation.
