# Supplementary material for: Effect of tofogliflozin on arterial stiffness in patients with type 2 diabetes: prespecified sub-analysis of the prospective, randomized, open-label, parallel-group comparative UTOPIA trial
Source: Cardiovasc Diabetol. 2021 Jan 4;20:4. doi: 10.1186/s12933-020-01206-1 (PMC7784389; doi:10.1186/s12933-020-01206-1)
Supplement: Supplementary file 6 — Additional file 6: Table S5. Effects of tofogliflozin on brachial-ankle pulse wave velocity in individuals with all three measurements (baseline, week 52, and week 104). [file 12933_2020_1206_MOESM6_ESM.docx]

**Additional file 6: Table S5. Effects of tofogliflozin on brachial–ankle pulse wave velocity in individuals with all three measurements (baseline, week 52, and week 104)**

|  | Tofogliflozin group  (n = 68) | Conventional group  (n = 60) | Treatment effect  (tofogliflozin–conventional treatment)  (mean change [95%CI]), p value | p value between groups |
| --- | --- | --- | --- | --- |
| Right baPWV |  |  |  |  |
| Baseline (cm/s) | 1731.1 ± 397.6 | 1687.4 ± 330.3 |  | 0.50 |
| Week 52 (cm/s) | 1732.4 ± 425.1 | 1745.5 ± 491.7 |  | 0.87 |
| Week 104 (cm/s) | 1667.7 ± 318.2 | 1735.1 ± 366.6 |  | 0.27 |
| Change from baseline at week 52 (cm/s) | 1.3 ± 234.5 | 58.0 ± 317.8 | –56.8 (–153.7, 40.2), p = 0.25 |  |
| Change from baseline at week 104 (cm/s) | –63.4 ± 251.7^*^ | 47.7 ± 221.1 | –111.1 (–194.5, 27.8), p = 0.009 |  |
| Left baPWV |  |  |  |  |
| Baseline (cm/s) | 1733.3 ± 435.4 | 1679.4 ± 349.1 |  | 0.45 |
| Week 52 (cm/s) | 1733.3 ± 459.7 | 1745.7 ± 494.0 |  | 0.48 |
| Week 104 (cm/s) | 1691.9 ± 390.3 | 1747.6 ± 364.0 |  | 0.41 |
| Change from baseline at week 52 (cm/s) | 0.0 ± 252.6 | 66.3 ± 302.8 | –66.4 (–163.5, 30.8), p = 0.18 |  |
| Change from baseline at week 104 (cm/s) | –41.4 ± 230.0 | 68.2 ± 231.2^*^ | –109.6 (–190.5, 28.8), p = 0.008 |  |
| Mean baPWV |  |  |  |  |
| Baseline (cm/s) | 1732.2 ± 410.4 | 1683.4 ± 332.7 |  | 0.47 |
| Week 52 (cm/s) | 1732.8 ± 439.9 | 1745.6 ± 489.4 |  | 0.88 |
| Week 104 (cm/s) | 1679.8 ± 348.8 | 1741.4 ± 362.0 |  | 0.33 |
| Change from baseline at week 52 (cm/s) | 0.6 ± 236.0 | 62.2 ± 304.7 | –61.6 (–156.3, 33.2), p = 0.20 |  |
| Change from baseline at week 104 (cm/s) | –52.4 ± 234.8 | 58.0 ± 214.7^*^ | –110.4 (–189.5, 31.3), p = 0.007 |  |

Data are presented as mean ± SD, unless stated otherwise. Comparisons of baPWV values during treatment with those at baseline were performed using a one-sample *t*-test based on the mixed-effects model for repeated measures. ^*^ p < 0.05, ^#^ p < 0.01, ^§^ p < 0.001. Differences in baPWV between groups at each point were analyzed using Student’s *t*-test. Differences in delta change in baPWV from baseline to weeks 52 and 104 between groups at each point (treatment effect) were analyzed using Student’s *t*-test. baPWV: brachial–ankle pulse wave velocity; SD: standard deviation.
