# Supplementary material for: Effect of tofogliflozin on arterial stiffness in patients with type 2 diabetes: prespecified sub-analysis of the prospective, randomized, open-label, parallel-group comparative UTOPIA trial
Source: Cardiovasc Diabetol. 2021 Jan 4;20:4. doi: 10.1186/s12933-020-01206-1 (PMC7784389; doi:10.1186/s12933-020-01206-1)
Supplement: Supplementary file 7 — Additional file 7: Table S6. Effects of tofogliflozin on brachial-ankle pulse wave velocity change in subgroups. [file 12933_2020_1206_MOESM7_ESM.docx]

**Additional file 7: Table S6. Effects of tofogliflozin on** **brachial–ankle pulse wave velocity change in subgroups**

| Subgroup |  | Mean change (±SD) from baseline to week 104 in Tofogliflozin group | Mean change (±SD) from baseline to week 104 in Conventional group | Treatment effect  (tofogliflozin–conventional treatment)  (mean change [95%CI]), p value |
| --- | --- | --- | --- | --- |
| Sex |  |  |  |  |
| Male | Right baPWV (cm/s) | –75.5 ± 280.3 (n = 42) | 51.2 ± 242.1 (n = 41) | -126.7 (-241.2, -12.2), P=0.031 |
|  | Left baPWV (cm/s) | –72.4 ± 265.2 (n = 42) | 58.4 ± 245.1 (n = 41) | -130.8 (-242.4, -19.2), P=0.022 |
|  | Mean baPWV (cm/s) | –73.9 ± 266.7 (n = 42) | 54.8 ± 230.9 (n = 41) | -128.7 (-237.8, -19.7), *P* = 0.021 |
| female | Right baPWV (cm/s) | –44.0 ± 200.6 (n = 26) | 48.1 ± 167.7 (n = 21) | -92.1 (-202.5, 18.2), P=0.10 |
|  | Left baPWV (cm/s) | 8.6 ± 148.9 (n = 26) | 82.0 ± 194.0 (n = 21) | -73.4 (-174.1, 27.3) *P* = 0.15 |
|  | Mean baPWV (cm/s) | –17.7 ± 170.5 (n = 26) | 65.1 ± 172.1 (n = 21) | -82.8 (-184.0, 18.4), *P* = 0.11 |
| Age |  |  |  |  |
| <65 years | Right baPWV (cm/s) | –18.1 ± 149.6 (n = 37) | 44.9 ± 189.8 (n = 31) | –63.0 (–145.2, 19.2), p = 0.13 |
|  | Left baPWV (cm/s) | –16.4 ± 142.8 (n = 37) | 44.8 ± 198.2 (n = 31)^*^ | –61.1 (–143.9, 21.6), p = 0.15 |
|  | Mean baPWV (cm/s) | –17.2 ± 139.6 (n = 37) | 44.8 ± 187.8 (n = 31) | –62.1 (–141.4, 17.3), p = 0.12 |
| ≧65 years | Right baPWV (cm/s) | –117.5 ± 330.2 (n = 31) | 55.5 ± 246.6 (n = 31) | –173.0 (–321.1, –25.0), p = 0.023 |
|  | Left baPWV (cm/s) | –71.4 ± 303.3 (n = 31) | 88.0 ± 255.3 (n = 31) | –159.4 (–301.8, –16.9), p = 0.029 |
|  | Mean baPWV (cm/s) | –94.4 ± 310.5 (n = 31) | 71.8 ± 235.1 (n = 31) | –166.2 (–306.1, –26.3), p = 0.021 |
| Current smoking |  |  |  |  |
| Yes | Right baPWV (cm/s) | –20.1 ± 87.2 (n = 13) | –12.1 ± 213.1 (n = 12) | –8.0 (–140.7, 124.7), p = 0.90 |
|  | Left baPWV (cm/s) | –9.5 ± 118.0 (n = 13) | –9.1 ± 173.8 (n = 12) | –0.5 (–122.5, 121.6), p = 0.90 |
|  | Mean baPWV (cm/s) | –14.8 ± 94.0 (n = 13) | –10.6 ± 188.9 (n = 12) | –4.2 (–126.2, 117.7), p = 0.94 |
| No | Right baPWV (cm/s) | –73.7 ± 276.3 (n = 55) | 65.1 ± 219.0 (n = 50)^*^ | –138.8 (–236.0, –41.7), p = 0.006 |
|  | Left baPWV (cm/s) | –49.0 ± 249.5 (n = 55) | 84.5 ± 236.7 (n = 50)^*^ | –133.5 (–227.8, –39.1), p = 0.006 |
|  | Mean baPWV (cm/s) | –61.3 ± 256.9 (n = 55) | 74.8 ± 214.9 (n = 50)^*^ | –136.1 (–228.3, –44.0), p = 0.004 |
| BMI |  |  |  |  |
| <25 kg/m^2^ | Right baPWV (cm/s) | –31.8 ± 184.9 (n = 31) | 21.1 ± 224.2 (n = 28) | –52.9 (–159.6, 53.8), p = 0.33 |
|  | Left baPWV (cm/s) | –6.5 ± 170.8 (n = 31) | 32.9 ± 185.3 (n = 28) | –39.4 (–132.2, 53.5), p = 0.40 |
|  | Mean baPWV (cm/s) | –19.1 ± 173.0 (n = 31) | 27.0 ± 197.4 (n = 28) | –46.1 (–142.7, 50.5), p = 0.34 |
| ≧25 kg/m^2^ | Right baPWV (cm/s) | –90.0 ± 296.3 (n = 37) | 74.1 ± 213.7 (n = 34) | –164.1 (–287.4, –40.8), p = 0.010 |
|  | Left baPWV (cm/s) | –70.7 ± 268.7 (n = 37) | 93.9 ± 256.9 (n = 34)^*^ | –164.7 (–289.4, –40.0), p = 0.010 |
|  | Mean baPWV (cm/s) | –80.4 ± 275.5 (n = 37) | 84.0 ± 221.8 (n = 34)^*^ | –164.4 (–283.5, –45.3), p = 0.008 |
| Duration of diabetes |  |  |  |  |
| <10 years | Right baPWV (cm/s) | –14.5 ± 146.8 (n = 31) | 132.8 ± 215.3 (n = 22)^#^ | 132.8 ± 215.3 (–247.0, –47.5), p = 0.005 |
|  | Left baPWV (cm/s) | –12.2 ± 144.6 (n = 31) | 143.8 ± 223.7 (n = 22)^#^ | –156.0 (–257.5, –54.5), p = 0.003 |
|  | Mean baPWV (cm/s) | –13.3 ± 139.1 (n = 31) | 138.3 ± 214.6 (n = 22)^#^ | –151.6 (–249.1, –54.1), p = 0.003 |
| ≧10 years | Right baPWV (cm/s) | –118.7 ± 319.4 (n = 34)^*^ | 3.5 ± 211.5 (n = 39) | –122.2 (–247.2, 2.8), p = 0.06 |
|  | Left baPWV (cm/s) | –75.2 ± 292.4 (n = 34) | 20.7 ± 223.2 (n = 39) | –95.9 (–216.4, 24.6), p = 0.12 |
|  | Mean baPWV (cm/s) | –97.0 ± 299.4 (n = 34) | 12.1 ± 200.9 (n = 39) | –109.1 (–226.7, 8.6), p = 0.07 |
| HbA1c |  |  |  |  |
| <7.0 % | Right baPWV (cm/s) | 35.7 ± 138.9 (n = 17) | 73.8 ± 196.1 (n = 19) | –38.1 (–154.6, 78.3), p = 0.51 |
|  | Left baPWV (cm/s) | 41.2 ± 106.8 (n = 17) | 76.4 ± 293.9 (n = 19) | –35.1 (–188.5, 118.2), p = 0.64 |
|  | Mean baPWV (cm/s) | 38.5 ± 117.8 (n = 17) | 75.1 ± 232.6 (n = 19) | –36.6 (–163.9, 90.6), p = 0.56 |
| ≧7.0 % | Right baPWV (cm/s) | –96.5 ± 272.5 (n = 51)^*^ | 39.7 ± 228.9 (n = 43) | –136.2 (–240.5, –32.0), p = 0.011 |
|  | Left baPWV (cm/s) | –69.0 ± 253.3 (n = 51) | 62.0 ± 195.5 (n = 43)^*^ | –131.0 (–225.0, –36.9), p = 0.007 |
|  | Mean baPWV (cm/s) | –82.7 ± 256.3 (n = 51)^*^ | 50.9 ± 203.9 (n = 43) | –133.6 (–229.7, –37.4), p = 0.007 |
| Hypertension |  |  |  |  |
| Yes | Right baPWV (cm/s) | –77.6 ± 250.0 (n = 29) | 29.3 ± 221.0 (n = 41) | –106.9 (–219.9, 6.1), p = 0.06 |
|  | Left baPWV (cm/s) | –64.8 ± 222.0 (n = 29) | 41.3 ± 242.8 (n = 41) | –106.1 (–219.6, 7.4), p = 0.07 |
|  | Mean baPWV (cm/s) | –71.2 ± 227.0 (n = 29) | 35.3 ± 219.2 (n = 41) | –106.5 (–214.2, 1.2), p = 0.05 |
| No | Right baPWV (cm/s) | –52.9 ± 255.6 (n = 39) | 91.0 ± 212.2 (n = 21) | –143.9 (–274.7, –13.0), p = 0.032 |
|  | Left baPWV (cm/s) | –24.0 ± 237.1 (n = 39) | 115.4 ± 190.9 (n = 21)^*^ | –139.4 (–259.8, –19.0), p = 0.024 |
|  | Mean baPWV (cm/s) | –38.5 ± 242.4 (n = 39) | 103.2 ± 192.5 (n = 21)^*^ | –141.6 (–264.3, –19.0), p = 0.024 |
| Dyslipidemia |  |  |  |  |
| Yes | Right baPWV (cm/s) | –87.8 ± 290.5 (n = 41) | 46.6 ± 214.8 (n = 41) | –134.4 (–246.7, –22.1), p = 0.020 |
|  | Left baPWV (cm/s) | –59.2 ± 264.6 (n = 41) | 47.1 ± 249.3 (n = 41) | 106.3 (–219.3, 6.7), p = 0.06 |
|  | Mean baPWV (cm/s) | –73.5 ± 271.6 (n = 41) | 46.9 ± 218.9 (n = 41) | –120.3 (–228.8, –11.9), p = 0.030 |
| No | Right baPWV (cm/s) | –26.5 ± 176.0 (n = 27) | 57.1 ± 230.3 (n = 21) | –83.7 (–201.6, 34.3), p = 0.16 |
|  | Left baPWV (cm/s) | –14.5 ± 165.4 (n = 27) | 104.0 ± 177.7 (n = 21)^*^ | –118.5 (–218.6, –18.4), p = 0.021 |
|  | Mean baPWV (cm/s) | –20.5 ± 163.9 (n = 27) | 80.6 ± 199.2 (n = 21) | –101.1 (–206.5, 4.4), p = 0.06 |
| Use of glucose-lowering agents |  |  |  |  |
| Yes | Right baPWV (cm/s) | –63.6 ± 267.5 (n = 58) | 44.2 ± 228.7 (n = 50) | –107.8 (–203.6, –12.1), p = 0.028 |
|  | Left baPWV (cm/s) | –42.2 ± 244.5 (n = 58) | 54.5 ± 239.1 (n = 50) | –96.7 (–189.3, –4.1), p = 0.041 |
|  | Mean baPWV (cm/s) | –52.9 ± 249.6 (n = 58) | 49.4 ± 222.5 (n = 50) | –102.3 (–193.1, –11.4), p = 0.028 |
| No | Right baPWV (cm/s) | –62.4 ± 134.9 (n = 10) | 75.1 ± 174.8 (n = 12) | –137.5 (–278.7, 3.7), p = 0.06 |
|  | Left baPWV (cm/s) | –36.9 ± 122.7 (n = 10) | 115.8 ± 172.0 (n = 12)^*^ | –152.7 (–288.3, –17.2), p = 0.029 |
|  | Mean baPWV (cm/s) | –49.7 ± 125.9 (n = 10) | 95.5 ± 159.5 (n = 12) | –145.1 (–274.9, –15.3), p = 0.030 |
| Use of ACEIs or ARBs |  |  |  |  |
| Yes | Right baPWV (cm/s) | –133.8 ± 268.5 (n = 17) | 47.4 ± 199.6 (n = 38) | –181.2 (–311.5, –50.9), p = 0.007 |
|  | Left baPWV (cm/s) | –111.6 ± 215.4 (n = 17)^*^ | 43.2 ± 244.8 (n = 38) | –154.7 (–293.0, –16.5), p = 0.029 |
|  | Mean baPWV (cm/s) | –122.7 ± 228.7 (n = 17)^*^ | 45.3 ± 212.7 (n = 38) | –168.0 (–295.4, –40.6), p = 0.011 |
| No | Right baPWV (cm/s) | –40.0 ± 244.0 (n = 51) | 54.6 ± 249.6 (n = 24) | –94.6 (–215.9, 26.7), p = 0.12 |
|  | Left baPWV (cm/s) | –18.0 ± 232.0 (n = 51) | 103.2 ± 197.1 (n = 24)^*^ | –121.2 (–230.5, –11.9), p = 0.030 |
|  | Mean baPWV (cm/s) | –29.0 ± 234.3 (n = 51) | 78.9 ± 212.2 (n = 24) | –107.9 (–220.2, 4.4), p = 0.06 |
| Use of statins |  |  |  |  |
| Yes | Right baPWV (cm/s) | –140.8 ± 316.5 (n = 28) | 81.3 ± 172.7 (n = 29)^*^ | –-222.1 (–356.8, –87.4), p = 0.002 |
|  | Left baPWV (cm/s) | –81.4 ± 294.1 (n = 28) | 91.2 ± 200.0 (n = 29)^*^ | –172.7 (–305.7, –39.6), p = 0.012 |
|  | Mean baPWV (cm/s) | –111.1 ± 300.2 (n = 28) | 86.3 ± 179.7 (n = 29)^*^ | –197.4 (–328.2, –66.6), p = 0.004 |
| No | Right baPWV (cm/s) | –9.3 ± 179.3 (n = 40) | 22.9 ± 251.2 (n = 33) | –32.2 (–132.8, 68.5), p = 0.53 |
|  | Left baPWV (cm/s) | –13.4 ± 170.5 (n = 40) | 44.5 ± 250.6 (n = 33) | –58.0 (–156.6, 40.7), p = 0.25 |
|  | Mean baPWV (cm/s) | –11.4 ± 167.6 (n = 40) | 33.7 ± 235.9 (n = 33) | –45.1 (–139.4, 49.3), p = 0.34 |

Data are presented as mean ± SD, unless stated otherwise. Comparisons of baPWV values during treatment with those at baseline were performed using a one-sample *t*-test based on the mixed-effects model for repeated measures. ^*^p < 0.05, ^#^ p < 0.01, ^§^p < 0.001.

Differences in delta change in baPWV from baseline to weeks 52 and 104 between groups at each point (treatment effect) were analyzed with the mixed-effects model for repeated measures. Treatment group, week, interactions between treatment group and week, age, sex, use of insulin at baseline, and baseline baPWV were included as fixed effects. baPWV, brachial-ankle pulse wave velocity; SD: standard deviation; ACEI, angiotensin-converting enzyme inhibitors; ARB, angiotensin II receptor blockers.
